# Effects of Key Farm Management Practices on Pullets Welfare—A Review

**DOI:** 10.3390/ani12060729

**Published:** 2022-03-14

**Authors:** Xiaohui Du, Pingwu Qin, Yanting Liu, Felix Kwame Amevor, Gang Shu, Diyan Li, Xiaoling Zhao

**Affiliations:** 1 College of Animal Science and Technology, Sichuan Agricultural University, Chengdu 611130, China; 10400@sicau.edu.cn (X.D.); qinpingwu1998@163.com (P.Q.); lyanting0212@163.com (Y.L.); amevorfelix@gmail.com (F.K.A.); diyanli@sicau.edu.cn (D.L.); 2 Department of Basic Veterinary Medicine, Sichuan Agricultural University, Chengdu 611130, China; dyysg2005@sicau.edu.cn; 3 Farm Animal Genetic Resources Exploration and Innovation Key Laboratory of Sichuan Province, Sichuan Agricultural University, Chengdu 611130, China

**Keywords:** welfare, pullet status, housing type, environmental management

## Abstract

**Simple Summary:**

Studies on animal behavior and welfare have reported that improving the management practices of pullets can enhance their growth, as well as their physical and mental condition, thus benefiting the productivity of laying hens. Therefore, in this review, we elaborated on the key effective farm management measures, including housing type and matching, flock status, and environmental management and enrichment, to provide the necessary information to incorporate welfare into chicken rearing and its importance in production, with the aim of improving the quantity and quality of chicken products.

**Abstract:**

Studies on animal behavior and welfare have reported that improving the management practices of pullets can enhance their growth, as well as their physical and mental condition, thus benefiting the productivity of laying hens. There is growing confidence in the international community to abandon the conventional practices of “cage-rearing and beak-trimming” to improve the welfare of chickens. Therefore, in this review, we summarized some of the effective poultry management practices that have provided welfare benefits for pullets. The results are as follows: 1. Maintaining similar housing conditions at different periods alleviates fear and discomfort among pullets; 2. Pullets reared under cage-free systems have better physical conditions and temperaments than those reared in cage systems, and they are more suitable to be transferred to similar housing to lay eggs; 3. Improving flock uniformity in appearance and body size has reduced the risk of pecking and injury; 4. Maintaining an appropriate population (40–500 birds) has reduced flock aggressiveness; 5. A combination of 8–10 h of darkness and 5–30 lux of light-intensity exposure via natural or warm white LED light has achieved a welfare–performance balance in pullets. (This varies by age, strain, and activities.); 6. Dark brooders (mimicking mother hens) have alleviated fear and pecking behaviors in pullets; 7. The air quality of the chicken house has been effectively improved by optimizing feed formulation and ventilation, and by reducing fecal accumulation and fermentation; 8. Complex environments (with litter, perches, straw bales, slopes, platforms, outdoor access, etc.) have stimulated the activities of chickens and have produced good welfare effects. In conclusion, the application of comprehensive management strategies has improved the physical and mental health of pullets, which has, in turn, improved the quantity and quality of poultry products.

## 1. Introduction

In recent years, animal welfare campaigns and studies have increased worldwide. A series of “group-rearing and untrimmed” strategies for poultry farming, combined with the selection of good-tempered pullets, the integrated application of various environmental enrichments, and the adoption of appropriate management measures, can effectively promote the welfare of chickens, especially at early ages [1,2]. Pullets with well-developed musculoskeletal and nervous systems easily adjust to various complex environmental changes, thereby significantly reducing the incidence of fear and pecking during laying (even if kept in traditional laying cages after rearing) [3,4], and they exhibit better health and production performance, thereby producing quality products [5,6,7]. Therefore, the aim of this review is to elaborate on key, effective farm management measures, focusing on three main aspects, including housing type and matching, flock status, and environmental management, to provide information needed to incorporate welfare into chicken rearing and to discuss its importance in production.

## 2. Housing Type and Matching

Currently, in poultry production, pullets are rearing in multilayer colony cages or cage-free houses, and they are later transferred to laying systems consisting of four options (cages, barns, aviaries, and outdoor ranges). Such a shift requires the flock to adapt to the new environment, which can be more or less stressful depending on the matching of different housing types. Major shortfalls and solutions to current laying housing systems and management practices that require immediate attention are discussed in these two rearing systems, as shown in Table 1.

### 2.1. Cage Rearing and Transfer Effects

Cage rearing system is the most predominant system of rearing chickens worldwide, in which birds are fed and watered in prefabricated cages with specific dimensions for efficient management [8,9,10].

The cage rearing system of keeping chickens is faced with key problems that affect the welfare of the birds. First, due to the lack of life experience in three dimensions, cage-raised pullets that lay eggs in aviaries are at a higher risk for feed waste, dehydration, and ground eggs [8], as basic needs (such as feed, water, perches, and nest boxes) are usually placed at different tiers in the aviary. Flocks without aviary experience are also prone to flight accidents after transition, resulting in keel fractures and a high rate of vent pecking [9]. In addition, chicks exposed to perches from hatching behaved better at moving between the layers of the coop at 16 weeks than those first exposed to perches at 8 weeks [10]. Delaying access to perches and laying nests until 25 weeks of age usually affects movement, use of vertical space, and the nocturnal falls of pullets for at least the next 10 weeks. Moreover, dust particles, management level, and total costs of aviaries are much higher than those of colony cages (including perches, dust bath mats, and laying boxes during the laying period) and traditional floor barns [11]. Therefore, it is important to avoid transferring cage-raised pullets to aviaries for laying in the production system. However, if necessary, the transition should be completed before 17 weeks of age with a supply of perches and laying nests [21,22].

Nevertheless, transferring cage-reared pullets to enriched colony cages for laying greatly reduces the discomfort, as well as the density and hardness of the tibial cortex of hens in the later laying period, over that of traditional cage-layers, indicating that the enriched cages provide opportunities for the hens to exercise, which enhance the development of bone-mass parameters [12].

### 2.2. Cage-Free Rearing and Transfer Effects

Compared with cage-reared pullets, cage-free-reared pullets show better adaptability and bone-bearing capacity [13], and they are usually transferred to one of the four current laying systems: battery cages, conventional barns, aviaries, or outdoor ranges.

Previous studies have shown that, in the first few days after the transition of pullets reared in an aviary to a laying system consisting of one of the four options (cages, barns, aviaries, and outdoor ranges), transfer results in crouching; however, there is no difference after 15 days of transition [23,24], which means hens habituate to new spaces in a relatively short period. Meanwhile, compared to the cage-reared birds, mortality in furnished cages caused by frustration and feather pecking is higher among aviary-reared pullets, suggesting that their later welfare may be compromised [14,25,26]; however, early transfer to a laying aviary (16 weeks or earlier) could reduce this effect [15]. In addition, pullets transferred from aviaries to enriched cages exhibit less fear, more dust bath activity, and use higher perches compared to cage-reared pullets [16,17]. Similarly, a study showed that birds reared in an aviary have higher laying rates in the nest, as compared to those reared in a barn-housed group [18].

Compared with the pullets kept indoors, outdoor-range-reared pullets showed improved growth and development because they were exposed to available natural light. The reason for this is that those pullets that are kept outdoors are free to exercise, which readies them to utilize the available outdoor area as adults and thus improve their health [19,27]. A study has achieved similar effects by adopting aviary or other enrichment methods during rearing to optimize the growth and development of pullets, and the follow-up effect of outdoor stocking. This could help pullets to adapt to the laying environment, thereby encouraging hens to lay eggs in their nests [20].

In summary, cage-free-reared pullets are raised with abundant environmental stimulation and provided with enough space for exercise, improving skeletal characteristics, reducing fear, and enhancing adaptability, compared to the cage-reared ones. Worldwide, we can find producers and technicians who need to be convinced about these systems. It is therefore recommended that pullets raised in cages should not be transferred to aviaries to lay eggs, but rather should preferably be transferred to enriched cages. Again, it is recommended that if the goal is to lay eggs in an aviary, it is better to rear pullets in an aviary. If aiming for free-range laying, it is better to provide pullets with the necessary outdoors experience as early as possible.

## 3. Flock Status

Good feather and physical condition of pullets are parameters that are used to predict high egg production and low mortality rates, and the welfare of pullets can be improved by ensuring flock uniformity, appropriate flock size, distribution, and stocking density [1,28].

### 3.1. Flock Uniformity

Flock uniformity includes consistency in body weight, feather color, and state. Studies have shown that preventing underweight conditions in pullets, increasing flock uniformity, and preventing pain and lameness among chickens could reduce the risk of pecking and cannibalism [15]. Furthermore, a study by Janczak et al. (2015), reported that Lohmann hens with a uniformity above 90% at 15 weeks old have a lower mortality rate during the laying period than flocks with uniformity rates between 85 and 90% or below 85%, whereas the average body weight at 15 weeks old had no effect on mortality [15]. In addition, a survey of 122 Canadian egg farms found that isolating chickens with abnormal conditions (such as being underweight, having messy or prominent plumage, or suffering from surface lesions or lameness) could reduce the risk of severe feather pecking and cannibalism (chickens pluck feathers from their companions for fiber rather than simply swallowing naturally shed feathers), as chickens with different appearances or behaviors are vulnerable to attack [29]. Therefore, the level of harmful pecking behavior can be significantly reduced through a combination of lighting, feeding, and flock adjustment [30], and a “no trimming” policy is expected to be incorporated into the sustainable egg-production system [31]. However, these management tasks are highly dependent on the conscientiousness and competence of the farm staff.

### 3.2. Flock Size and Distribution

Since social animals usually behave synchronously, resources should be allocated on the basis of flock size, stocking density, and behavior, especially in non-cage production systems. Several studies on the effects of feather pecking and aggression on the social behavior of pullets and laying hens have shown that aggressive pecking is most common in smaller flocks, possibly because these birds usually form social hierarchies, whereas birds in larger groups often have trouble distinguishing between familiar and unfamiliar companions and then develop tolerance strategies, so they tend to be less aggressive [32,33,34]. Furthermore, with increasing group size, chickens became less vigilant, with more hens resting on the lower perch [35], fewer birds roosting on higher perches, and more birds engaging in preening on the floor [36]. In cage-free systems, smaller flocks tend to have a higher proportion of outdoor activities [37], which can reduce pecking injuries. Therefore, the flock size should be controlled at fewer than 500 [38,39] and more than 40 individuals [32,33,34].

A study on the behavioral synchronization and spatial clustering of commercial pullets reported that the relative synchronization degree of ingestion, drinking water, resting, and preening declined exponentially as flock size (5–120 individuals per group) increased although the absolute number of pullets increased. Among these behaviors, preening was the most synchronous behavior (more than twice that of the least synchronized behavior), and feeding was the most clustered behavior in space (three times more clustered than the other behaviors) [40]. This demonstrates the importance of providing sufficient space (especially feeding space) for individual birds, which is more important in small flocks (more synchronous) than in large or cage-free flocks.

In general, ensuring that all pullets are occupied during the day, as well as avoiding their pecking at each other, is very important to improve the welfare of pullets, and this can be achieved by adopting proper housing systems and using the correct stocking density.

### 3.3. Stocking Density

Stocking density can influence the health condition and adaptability of pullets during the rearing stage. Usually, decreased stocking density and the provision of proper environmental enrichment can reduce plumage damage. The maximum suitable density of 16-week-old laying pullets is 11–14 per m^2^ without environmental enrichment, and it is important to adjust the density required by different breeds of chickens through behavioral observation [41]. In the presence of enrichment (pecking stones, pecking blocks, and alfalfa bales), the stocking density can be properly increased, and the increasing effect can be better than simply decreasing the stocking density (from 22 to 17 pullets per m^2^) [42,43]. Crowded pullets exhibit more anxious behaviors and elevated corticosterone levels in their plasma and feathers, which could impair the adaptability of the pullets and cause long-term, adverse consequences [44].

In addition, studies have shown that different hens occupy different spaces when expressing different behaviors. On average, compared with a white hen, a brown hen needs 89.6 cm^2^ more space when standing, 81.5 cm^2^ more space when lying, 572 cm^2^ more space when flapping, 170.3 cm^2^ more space when dust bathing, and 3.38 cm more length while perching. Hens of all strains were wider when roosting than the recommended 15 cm per hen. Therefore, various factors (including breed type, body size, flock size, stocking density, environmental management and enrichment, facility layout, and synchronicity) should be comprehensively considered in the development of industry guidelines and regulations [45,46].

## 4. Environmental Management

Chickens are naturally disposed to fearfulness and sensitivity, whereas young red jungle fowls exhibit fear responses and flight behavior for a few days post-hatch [47]. The peak for fear responses exhibited by chicks is within 10 days of hatching, and this is due to their visual development and novelty evaluation [48,49]. Stress reduces feed intake and growth and impairs immune response and function, resulting in high disease susceptibility [50]. Therefore, appropriate environmental management in production systems is beneficial for alleviating fear and stress among chickens.

### 4.1. Lighting Management

Light is an important environmental factor which could influence the behavior, growth, productivity, and welfare of poultry via three characteristics based on the natural photoperiod: duration, intensity, and color/wavelength, with each consisting of multiple practices. This makes the use of an artificial lighting regimen a popular and complex option for manipulating laying hen production; however, few studies have been published on the important early stages of laying hens [51,52,53]. With the emergence of multiple housing types and the current accumulation of knowledge of chicken behavior, early light management of pullets is worth attempting, through adjustments of the three aspects with the concern of the chicken’s welfare in mind.

#### 4.1.1. Light Duration

Light duration is important for the growth, reproduction, and welfare of chickens. In poultry production, chickens are confronted with long-light phases. However, extremely long-light cycles are associated with reduced performance. In the European Union, an uninterrupted darkness of 8 h for laying hens is mandatory to maintain normal circadian rhythms and promote maximum rest because intermittent darkness may affect the rest and feeding of the chickens, resulting in a variety of metabolic and immune disorders [15,54]. Hence, keeping chickens under short-light conditions could lead to stronger responsiveness against bacterial infections and better responses to vaccinations [53], and decrease the risk of vent pecking [55]. Given the freedom to choose different light intensities (<1 lux–100 lux), W-36 laying hens (23–30 weeks) spent an accumulation of 10.0 h in darkness (<1 lux) per day, and dark hours were distributed intermittently throughout the day, which differed from the typical commercial practice of providing continuous dark periods for certain parts of the day (e.g., 8 h at night) [56].

On the contrary, pullets reared on long durations (14 h to 17 h) mature faster than birds reared on constant 10 h [57]. Introducing 2 h midnight lighting (ML, 2 h + 12 h) late in the growing period (12–18 wk) also induced early maturity and had the least egg production (302) from 18 to 70 wk, whereas providing 2 h ML from 0–18 wk resulted in the greatest number of eggs (317), and ML given only from 0–12 wk of age had the effect of delaying maturity and produced a middling number of eggs (310–312) [58]. However, the midnight lighting treatments had quite minor effects on the growth and feed intake of pullets according to another study by the same authors [59].

#### 4.1.2. Light Intensity

Light intensity may affect laying hen behaviors and production performance. For caged, laying hen production, the dominant light intensity (LI) regime is 20 lux in the early stage, 5–10 lux in the growing stage, and 10–15 lux in the laying period [52].With the concerns of behavior and welfare, a study revealed that pullets spent more time preening at 50 lux than at 10 lux, spent more time wall pecking at 10 lux than at 50 lux, and had higher jumping frequency at 30 lux than at 10 lux [60]. When exposed to extremely high LI (500 lux [61], 121.8 lux [62]), layers produced smaller eggs in size and total egg mass, which indicated inadequate feed intake under high LI conditions. When exposed to low LI (1, 5, 11.9), a reduced rate of egg production resulted [61,62]. Given the freedom to choose LI, W-36 laying hens (23–30 weeks) generally spent more time in lower LI per day (an accumulation of 10.0 h at <1 lux, 6.4 h at 5 lux, 3.0 h at 15 lux, 3.1 h at 30 lux, and 1.5 h at 100 lux), and ingested the highest amount of feed at 5 lux (28.4 g/hen, 32.5% daily total) and the lowest amount at 100 lux (5.8 g/hen, 6.7%) [56]. The above studies revealed that pullets generally preferred a different intensity of light for different activities; and it suggested providing light intensities varying between 5 and 30 lux at different locations to achieve welfare–performance balance.

#### 4.1.3. Light Color/Wavelength

Birds can perceive colored light (400–700 nm) as well as the ultraviolet (UV) portion of the spectrum (100–400 nm) due to the presence of external retinal cones in their eyes [63]. Light colors may affect poultry behaviors, well-being, and performance and produce different results. Current mainstream light-emitting diode (LED) lights are better than incandescent lamps and fluorescent light in energy savings, longevity, and other aspects [64]. With LED lamps widely applied in poultry housing systems, specific light colors (white, red, blue, green, or combinations) have been investigated as an additional management tool to achieve better performance and welfare of chickens [53].

Studies have confirmed that pullets exposed to longer wavelengths of light (LWL, red/yellow/orange) have increased egg production later in life compared to shorter wavelengths of light (SWL, blue/green) although responses may vary depending on bird strain and the intensity of the light used [63,65]. In addition, it was monitored that follicle-stimulating hormone (FSH) concentration, ovarian weight, and follicle number increased in hens raised under LWL [66]. The reason is that LWL contains more energy and can penetrate deeper tissues more easily, thus, releasing more gonadotropin-releasing hormone (GnRH) and FSH, increasing egg production. Some researchers have speculated that SWL plus higher intensities may produce the same effect as red light, which has been demonstrated to stimulate an increase of luteinizing hormone (LH) in quails [67].

It was reported that increasing the intensity at shorter wavelengths can compensate for differences in light-color effects. Moreover, SWL could reduce the activities and fear responses of chickens, with younger chickens being more sensitive to green light, whereas older chickens are more sensitive to blue light [68]. Moreover, providing layer pullets (54–82 d) freedom to stay under 4 LED color lights, they preferred blue light the most and red light the least [69], and most pullets preferred to drink under the blue and white lights [70]. This is understandable because chicks in their natural state often stay under hens’ wings and rarely encounter red lights. (See the following section on the dark brooder). Based on the above studies, Wei et al. (2020) exposed chickens to combinations of LED lights (white light (400–700 nm, WL), blue/green (435–565 nm, BG), and yellow/orange (565–630 nm, YO), in the following patterns: BG at 1 D–13 wk + YO at 14–20 wk (BG–YO)) on pullets from 1 D to 20 wk, and revealed that, compared with the other treatments, the YO treatment significantly increased the bone-mineral density of the layer (*p* < 0.05) and reached 50% egg production age first; BG–YO treatment promoted the development of the sexual organs (oviducts and ovaries) of the laying hens at the age of 20 wk (*p* < 0.05); the BG–YO and BG treatment had higher serum Ig concentrations at 13 wk of age (*p* < 0.05) [65]. The results are consistent with the above studies, suggesting and demonstrating that appropriately using LED lights during brooding and rearing periods could have positive effects on the immune performance, bone development, and later production performance of pullets.

Currently, transforming the color of the light to red or dimming the light has been regarded as an effective method to alleviate the fear responses and reduce the risk of feather pecking in layers and mortality from cannibalism because it reduces the birds’ ability to recognize blood and bare skin [71]. However, the red light may interfere with other wavelengths penetrating the eyes [72], or dim light may not provide enough stimulation to develop the reproduction system of the pullets. Therefore, red light is best used as a short-term, curative measure, and not as a long-term, preventive measure, while green or blue light could have some comforting effects based on the age of birds [68,73]. However, further studies are needed to confirm the impact of color LEDs as a production practice on pullets.

Chickens can sense UV light [74], and natural-light-reared pullets showed a preference for natural light [19] and natural-like light (white LEDs or red + green + blue LEDs to match the forest understory spectra, 4500 K, with UV) were associated with more active behaviors and better plumage in laying hens, compared with artificial commercial lighting (warm white LED, 3000 K, no UV) [75]. The benefits of UV light are mediated through its ability to activate cholecalciferol from 7-dehydroxycholesterol in the skin, resulting in improvements in eggshell quality [63]. However, providing sunlight in addition to standard lighting [76,77] or increasing UV illuminance during the laying period could increase the risk of pecking because of excessive light intensity [78]. However, lack of UV light can negatively affect basal corticosterone levels and exploration [79,80], encouraging feather damage and cannibalism [81].

In conclusion, light duration and intensity are the most important factors that impact the development, activity, production, and welfare of chickens. This is due to the fact that light intensity can compensate for short-wave lights (blue/green) to achieve similar effects of long-wave lights (red/yellow/orange). Pullets can achieve welfare–performance balance with a guaranteed 8–10 h of darkness, 5–30 lux light intensity exposed by natural-like or warm white LED (which varies by age, strain, and activities). Red or dim light is a short-term measure to prevent pecking addiction, whereas short-wave lights have some comforting benefits on pullets, but this requires further study.

### 4.2. Dark Brooder

In poultry production, chicks and hens are usually separated, leading to high levels of stress, fear, crowding, suffocation, injury, and pecking [82]; especially, young free-range flocks are prone to panic [83].

The presence of hens during the rearing period has an important influence on the behavioral development of chicks, such as increasing their foraging activity, reducing fear [84], and avoiding danger [85,86]. During the first week, the brooding hens pecked the ground four times as much as their chicks, presumably stimulating pecking behavior. Such pecking did not result in a higher feather pecking frequency because the chicks were guided by the hens to peck at materials such as feed and bedding materials [87]. Pullets with hen-care during their first 53 days of life are less fearful and more socially motivated between 14 and 29 weeks of age than non-brooded pullets [88]. In addition, brooding hens guide their chicks to roosting earlier (3.5 days earlier), which reduces the risk of pecking due to ground congestion [87].

Chicks are more comfortable when resting under the dark wings of hens, but the development of hen-raising systems that do not endanger animal health or efficiency is a challenge for research and industry. Hence, the creation of dark brooders (DB, mimicking hens) with heating and shading effects was introduced. Studies have shown that chicks of laying hens raised in DB were similar to the effect of brood hens [89,90], with significant synchronous activities (longer active period and resting period) [91], better feathers and skin conditions at 23 of weeks age (greatly reducing feather pecking frequency), and lower mortality [87,92]. Such effects have been reported in commercial chicken production, from 1 to 35 weeks old, and the pecking rate of DB chickens was significantly lower than that of the control group, while the feather condition was improved with no adverse effects on the growth rate, weight uniformity, or production performance [73,93].

In conclusion, dark brooders can better simulate the brooding effect of hens, reduce fear and pecking among chicks, have no adverse effects on production performance, and have good commercial applications.

### 4.3. Manure Management

Harmful gases inevitably accumulate in intensive chicken farms and interact with other environmental components, especially at the stage of sensitive chicks with reduced ventilation during the cold season [53]. Continuous exposure to high concentrations of harmful gases can impair the immune system of chickens (resulting in widespread and secondary infections) [94], behavioral capacity [77], production characteristics, and the death of chickens [95,96]. Ultimately, this could cause acidification and eutrophication of ecosystems, which could harm human beings [97].

Usually, the air pollution of chicken houses is mainly caused by NH_3_ emission produced by urease degradation of uric acid in feces [98,99]. Therefore, manure management (especially ammonia control) is a major concern in the poultry industry. The European Union established Directive 2010/75/EU, and some developed countries have conducted inventories and measurements of odorants emitted from animal farms [100,101]. In addition, NH3 emissions usually increase under warm and wet conditions and worsen due to global warming [97].

Studies have shown that NH3 concentrations are generally high and exceed 20 ppm (the maximum allowable concentration in European countries) in litter-based housing types, including floor housing (66–120 ppm) and aviary systems (21–42 ppm), and low concentrations in furnished cages (3–12 ppm) [95,102]. Moreover, a study showed that human exposure to ammonia concentration associated with significant pulmonary function decrements was 12 ppm [103]. Therefore, under different circumstances, it is particularly important to maintain an ammonia concentration below 10–20 ppm in a chicken house [95,101]. Studies have revealed three main series of methods that can effectively reduce ammonia emissions in chicken farms: optimizing feed formula and additives to reduce uric acid excretion; proper drying, collection, or removal of poultry droppings from the chicken house in time to avoid uric acid decomposition; and centrally decomposing poultry droppings with manure additives.

Since uric acid excreted by birds is highly related to undigested proteins, it is essential to reduce the amount of uric acid in feces by avoiding overfeeding chickens with protein and improving their digestive capacity. Therefore, addition of essential amino acids (such as lysine and methionine) to substitute part of the protein, is a practical approach for reducing the protein level of diets. Furthermore, the addition of feed additives (probiotics) [104], wheat bran [105], and enzymes [106] (phytase, xylanase, and proteases) can promote feed digestibility.

To avoid accumulation and decomposition of feces in the chicken houses, a variety of methods for timely management of manure, such as the timely removal of feces by belts, scrapers, catchers, or other technological equipment [98]; replacing old litter with dry (dust-free), clean, fluffy, and mildew-free material; improving barn ventilation while maintaining an appropriate ambient temperature, or pump in heated air before ventilation in winter [97], and avoiding decomposition by keeping stacked manure dry in chicken houses (using fans, no leakage on waterline) [95].

Chicken manure is usually centrally collected and processed in special processing rooms, with continuous inoculation of probiotics to decompose feces at an appropriate temperature and humidity [107]. Moreover, spraying on or mixing additives (bentonite, sugarcane bagasse, and saline additives) with litter can reduce air pollution in chicken farms [108,109].

Therefore, manure management is an important factor in promoting quality air in the poultry industry, and an optimal solution can be found through comprehensive measures, such as optimizing feed formulation, manure ventilation, collection and removal facilities, and manure fermentation technology.

### 4.4. Complex Environment (CE)

In addition to the above environmental factors, there are other combinations of facilities that pullets have a high incentive to enjoy, collectively termed ‘complex environments’ (CE, with perches, litter, dark brooders, straw bales, slopes, platforms, outdoor access, etc.). CE has profound and long-lasting benefits for the welfare and stress adaptation of chickens, especially in the early stage. Studies have revealed that aviary-reared birds have low levels of fearfulness and use elevated areas of the pen more often compared with cage-reared birds [110], and CE (perches and dark brooder) birds had a higher resting behavior and more optimistic response (better resilience), approached ambiguous cues more quickly, and had lower heterophil/lymphocyte ratios after stressful challenges than birds reared in a simple environment [111]. Furthermore, when subjected to a predator test at 42 days of age, chicks (from one day old) reared in CE (perches and litter materials) were characterized by decreased fearfulness, lower plasma corticosterone, improved gut microbial functions, lower relative mRNA expression of GR, and elevated mRNA expressions of stress-related genes CRH, BDNF, and NR2A in the hypothalamus, compared to a litter-materials group or a barren environment group, thus enabling CE birds to comfortably cope with any future challenge [112].

Of all the CE, litter is considered unimportant, because it is prone to accumulating feces, as well as encouraging the spread of diseases, thereby prompting farmers to adopt the cage-rearing system. However, studies have shown that ectoparasite significantly increases preen behavior in caged chickens due to the lack of litter material, resulting in messy plumage, skin lesions, anemia, slow growth, lower egg production, and higher chances of being pecked [42,113,114]. Recently, studies have reported that changes in intestinal flora affect neurological diseases (anxiety, depression, etc.) through neural or hormonal pathways, and environmental enrichment in early life can affect adult behaviors, stress physiology [1,115], musculoskeletal and neurological development, health, egg quality, and other long-term benefits of pullets through the brain–gut axis, which could improve its response capacity in complex environments [116]. Therefore, litter material may have profound effects by establishing certain connections with nerves or hormones through the gut flora.

Chickens are selective about bedding material, depending on their physiological status and the behavior of their companions. They preferred materials such as peat, sand, and wood chips (which easily seeped into their feathers) for dustbathing, and preferred straws (long straws are better than short straws) for foraging [117,118]. Even in cage rearing, the provision of paper to chicks from day 1 led to less feather damage and fear at 30 wks than their counterparts [119,120]. Partially or completely removing chicken paper from the cage without providing other foraging materials caused the foraging behavior of the chicks to decrease, and the frequency of severe pecking to increase [121]. Therefore, the provision of appropriate litter materials and litter quality is of great significance for promoting foraging, dustbathing, and reducing pecking injuries among chickens.

## 5. Conclusions

In general, the welfare of pullets has been enhanced by several management practices, such as rearing pullets under cage-free systems, ensuring proper bird’s status and proper flock size (40–500 birds), providing 8–10 h of darkness and 5–30 lux of different light-intensity exposure via natural-like LED lights, furnishing appropriate complex environments (litter, perches, dark brooders, straw bales, slopes, platforms, etc.), improving the air quality of the chicken farm through good manure management. However, using appropriate management systems to promote the welfare of birds is complex due to the costs of materials, different chicken strains, ages, and diverse activities, as well as different housing conditions and seasons in different geographical locations. Therefore, there is a need for intensive education and the development of affordable strategies that will aid chicken farmers to ensure proper management practices by making use of any available resources to meet the welfare requirements of pullets and improve productivity.

## Figures and Tables

**Table 1:** The matching effects of different housing types on transfers from rearing to laying.

Rearing Type	Housing Conversion	Matching Effects	References
Cage rearing	To aviaries	Higher risk of feed waste, dehydration, and ground eggs	Tauson 2005 [8]
	To aviaries	Prone to flight accidents, keel fractures, and vent pecking	Gunnarsson et al., 1999 [9]
	To perches	chicks exposed to perches earlier behaved better at moving between the layers later.	Gunnarsson et al., 2000 [10]
	To floor barns with perch	Delaying access to perches for at least 10 weeks	Mitchell et al., 2015 [11]
	To enriched colony cages *	Reduces discomfort, enhances the development of bone mass parameters better than those of the traditional cage layers	Regmi et al., 2016 [12]
Cage-free rearing	From aviaries or cages to the same housing type or enriched cages	Total medullary and pneumatic bone weight and ash content scores from high to low were A-A, C-E, A-C and C-C hens, respectively	Neijat et al., 2019 [13]
	From aviaries to furnished cages at 16 weeks	Mortality (20–76 wk) is higher (5.52% vs. 2.48%) than cage-reared birds	Tahamtani et al., 2014 [14]
	From aviaries to cages	Early transfer (16 weeks or earlier) could reduce mortality and increase nest eggs	Janczak et al., 2015 [15]
	From aviaries to enriched cages *	Fewer acceleration events and collisions during daytime at 21 and 35 weeks of age, and more high-perching compared to conventional cages	Pulin et al., 2020 [16]
	From aviaries to enriched cages at 16 weeks *	Lower levels of fearfulness indicated by spending less time away from the novel object at 19 and 21 weeks compared to conventional cages	Brantsæter et al., 2016 [17]
	From aviaries to * aviaries	More eggs in the nest compared to barn-reared hens	Colson et al., 2008 [18]
	To outdoor *	The high outdoor hens showed the highest spleen and empty gizzard weights	Md Saiful et al., 2020 [19]
	To modified cages (with 2 nests each) *	Expressed a full repertoire of pre-laying activities; displacement behaviors and pacing were less frequent; more eggs in the nest than conventional cages without nests	Shervin et al., 1993 [20]

* Indicates the best matching effects.

## Data Availability

Not applicable.

## References

[1] Campderrich I., Nazar F.N., Wichman A., Marin R.H., Estevez I., Keeling L.J. (2019). Environmental complexity: A buffer against stress in the domestic chick. PLoS ONE.

[2] Rodenburg T.B., Van Krimpen M.M., De Jong I.C., De Haas E.N., Kops M.S., Riedstra B.J., Nicol C.J. (2013). The prevention and control of feather pecking in laying hens: Identifying the underlying principles. World’s Poult. Sci. J..

[3] de Haas E.N., Bolhuis J.E., Kemp B., Groothuis T.G., Rodenburg T.B. (2014). Parents and early life environment affect behavioral development of laying hen chickens. PLoS ONE.

[4] Mellor E., Brilot B., Collins S. (2018). Abnormal repetitive behaviours in captive birds: A Tinbergian review. Appl. Anim. Behav. Sci..

[5] Rubolini D., Romano M., Boncoraglio G., Ferrari R.P., Martinelli R., Galeotti P. (2005). Effects of elevated egg corticosterone levels on behavior, growth, and immunity of yellow-legged gull (*Larus michahellis*) chicks. Horm. Behav..

[6] Henriksen R., Groothuis T.G., Rettenbacher S. (2011). Elevated plasma corticosterone decreases yolk testosterone and progesterone in chickens: Linking maternal stress and hormone-mediated maternal effects. PLoS ONE.

[7] Henriksen R., Rettenbacher S., Ton G.G.G. (2013). Maternal corticosterone elevation during egg formation in chickens (*Gallus gallus domesticus*) influences offspring traits, partly via prenatal undernutrition. Gen. Comp. Endocrinol..

[8] Tauson R. (2005). Management and housing systems for layers—Effects on welfare and production. World’s Poult. Sci. J..

[9] Gunnarsson S., Keeling L.J., Svedberg J. (1999). Effect of rearing factors on the prevalence of floor eggs, cloacal cannibalism and feather pecking in commercial flocks of loose housed laying hens. Br. Poult. Sci..

[10] Gunnarsson S., Yngvesson J., Keeling L.J., Forkman B. (2000). Rearing without early access to perches impairs the spatial skills of laying hens. Appl. Anim. Behav. Sci..

[11] Mitchell D., Arteaga V., Armitage T., Mitloehner F., Tancredi D., Kenyon N. (2015). Cage Versus Noncage Laying-Hen Housings: Worker Respiratory Health. J. Agromed..

[12] Regmi P., Smith N., Nelson N., Haut R.C., Orth M.W., Karcher D.M. (2016). Housing conditions alter properties of the tibia and humerus during the laying phase in Lohmann white Leghorn hens. Poult. Sci..

[13] Neijat M., Casey-Trott T.M., Robinson S., Widowski T.M., Kiarie E. (2019). Effects of rearing and adult laying housing systems on medullary, pneumatic and radius bone attributes in 73-wk old Lohmann LSL lite hens1. Poult. Sci..

[14] Tahamtani F.M., Hansen T.B., Orritt R., Nicol C., Moe R.O., Janczak A.M. (2014). Does rearing laying hens in aviaries adversely affect long-term welfare following transfer to furnished cages?. PLoS ONE.

[15] Janczak A.M., Riber A.B. (2015). Review of rearing-related factors affecting the welfare of laying hens. Poult. Sci..

[16] Pullin A.N., Temple S.M., Bennett D.C., Rufener C.B., Blatchford R.A., Makagon M.M. (2020). Pullet Rearing Affects Collisions and Perch Use in Enriched Colony Cage Layer Housing. Animals.

[17] Brantsæter M., Tahamtani F.M., Moe R.O., Hansen T.B., Orritt R., Nicol C. (2016). Rearing Laying Hens in Aviaries Reduces Fearfulness following Transfer to Furnished Cages. Front. Vet. Sci..

[18] Colson S., Arnould C., Michel V. (2008). Influence of rearing conditions of pullets on space use and performance of hens placed in aviaries at the beginning of the laying period. Appl. Anim. Behav. Sci..

[19] Bari M.S., Laurenson Y.C., Cohen-Barnhouse A.M., Walkden-Brown S.W., Campbell D.L. (2020). Effects of outdoor ranging on external and internal health parameters for hens from different rearing enrichments. PeerJ.

[20] Sherwin C.M., Nicol C.J. (1993). A descriptive account of the pre-laying behaviour of hens housed individually in modified cages with nests. Appl. Anim. Behav. Sci..

[21] Ali B.A., Toscano M., Siegford J.M. (2019). Later exposure to perches and nests reduces individual hens’ occupancy of vertical space in an aviary and increases force of falls at night. Poult. Sci..

[22] MacLachlan S.S., Ali A.B.A., Toscano M.J., Siegford J.M. (2019). Influence of later exposure to perches and nests on flock level distribution of hens in an aviary system during lay. Poult. Sci..

[23] Faure J.M. (1991). Rearing conditions and needs for space and litter in laying hens. Appl. Anim. Behav. Sci..

[24] Craig J.V., Okpokho N.A., Milliken G.A. (1988). Floor- and cage-rearing effects on pullets’ initial adaptation to multiple-hen cages. Appl. Anim. Behav. Sci..

[25] Nicol C.J., Caplen G., Edgar J., Browne W.J. (2009). Associations between welfare indicators and environmental choice in laying hens. Anim. Behav..

[26] Nicol C.J., Caplen G., Edgar J., Richards G., Browne W.J. (2011). Relationships between multiple welfare indicators measured in individual chickens across different time periods and environments. Anim. Welf..

[27] Grigor P.N., Hughes B.O., Appleby M.C. (1995). Effects of regular handling and exposure to an outside area on subsequent fearfulness and dispersal in domestic hens. Appl. Anim. Behav. Sci..

[28] Milisits G., Szász S., Donkó T., Budai Z., Almási A., Pőcze O. (2021). Comparison of Changes in the Plumage and Body Condition, Egg Production, and Mortality of Different Non-Beak-Trimmed Pure Line Laying Hens during the Egg-Laying Period. Animals.

[29] Decina C., Berke O., van Staaveren N., Baes C.F., Widowski T.M., Harlander-Matauschek A. (2019). An Investigation of Associations Between Management and Feather Damage in Canadian Laying Hens Housed in Furnished Cages. Animals.

[30] Lambton S.L., Nicol C.J., Friel M., Main D.C.J., McKinstry J.L., Sherwin C.M. (2013). A bespoke management package can reduce levels of injurious pecking in loose-housed laying hen flocks. Vet. Rec..

[31] Kaukonen E., Valros A. (2019). Feather Pecking and Cannibalism in Non-Beak-Trimmed Laying Hen Flocks—Farmers’ Perspectives. Animals.

[32] Campderrich I., Liste G., Estevez I. (2017). Group size and phenotypic appearance: Their role on the social dynamics in pullets. Appl. Anim. Behav. Sci..

[33] Buchwalder T., Huber-Eicher B. (2005). Effect of group size on aggressive reactions to an introduced conspecific in groups of domestic turkeys (*Meleagris gallopavo*). Appl. Anim. Behav. Sci..

[34] Nicol C.J., Gregory N.G., Knowles T.G., Parkman I.D., Wilkins L.J. (1999). Differential effects of increased stocking density, mediated by increased flock size, on feather pecking and aggression in laying hens. Appl. Anim. Behav. Sci..

[35] Bilcík B., Keeling L.J. (2000). Relationship between feather pecking and ground pecking in laying hens and the effect of group size. Appl. Anim. Behav. Sci..

[36] Newberry R.C., Estevez I., Keeling L.J. (2001). Group size and perching behaviour in young domestic fowl. Appl. Anim. Behav. Sci..

[37] Gilani A.M., Knowles T.G., Nicol C.J. (2014). Factors affecting ranging behaviour in young and adult laying hens. Br. Poult. Sci..

[38] Hegelund L., Sørensen J.T., Kjaer J.B., Kristensen I.S. (2005). Use of the range area in organic egg production systems: Effect of climatic factors, flock size, age and artificial cover. Br. Poult. Sci..

[39] Bestman M.W.P., Wagenaar J.P. (2003). Farm level factors associated with feather pecking in organic laying hens. Livest. Prod. Sci..

[40] Keeling L.J., Newberry R.C., Estevez I. (2017). Flock size during rearing affects pullet behavioural synchrony and spatial clustering. Appl. Anim. Behav. Sci..

[41] Spindler B., Clauss M., Briese A., Hartung J. (2013). Planimetric measurement of floor space covered by pullets. Berl. Munch. Tierarztl. Wochenschr..

[42] Liebers C.J., Schwarzer A., Erhard M., Schmidt P., Louton H. (2019). The influence of environmental enrichment and stocking density on the plumage and health conditions of laying hen pullets. Poult. Sci..

[43] Zepp M., Schwarzer A., Helmer F., Louton H., Erhard M., Schmidt P. (2018). The influence of stocking density and enrichment on the occurrence of feather pecking and aggressive pecking behavior in laying hen chicks. J. Vet. Behav..

[44] von Eugen K., Nordquist R.E., Zeinstra E., van der Staay F.J. (2019). Stocking Density Affects Stress and Anxious Behavior in the Laying Hen Chick During Rearing. Animals.

[45] Riddle E.R., Ali A.B.A., Campbell D.L.M., Siegford J.M. (2018). Space use by 4 strains of laying hens to perch, wing flap, dust bathe, stand and lie down. PLoS ONE.

[46] Zimmerman P.H., Lindberg A., Pope S.J., Glen E., Bolhuis J., Nicol C.J. (2006). The effect of stocking density, flock size and modified management on laying hen behaviour and welfare in a non-cage system. Appl. Anim. Behav. Sci..

[47] Kruijt J.P. (1964). Ontogeny of Social Behaviour in Burmese Red Junglefowl (Callus Gallus Spadìceus) Bonnaterre.

[48] Campbell D.L.M., de Haas E.N., Lee C. (2019). A review of environmental enrichment for laying hens during rearing in relation to their behavioral and physiological development. Poult. Sci..

[49] Andrew R.J., Brennan A. (1983). The lateralization of fear behaviour in the male domestic chick: A developmental study. Anim. Behav..

[50] Abo-Al-Ela H.G., El-Kassas S., El-Naggar K., Abdo S.E., Jahejo A.R., Al Wakeel R.A. (2021). Stress and immunity in poultry: Light management and nanotechnology as effective immune enhancers to fight stress. Cell Stress Chaperones.

[51] Ray A., Pradhan R.K. (2012). Importance of Light in Poultry Industry. Res. J. Sci. Technol..

[52] Manser C.E. (1996). Effects of lighting on the welfare of domestic poultry: A review. Anim. Welf..

[53] Hofmann T., Schmucker S.S., Bessei W., Grashorn M., Stefanski V. (2020). Impact of Housing Environment on the Immune System in Chickens: A Review. Animals.

[54] Hieke A.S.C., Hubert S.M., Athrey G. (2019). Circadian disruption and divergent microbiota acquisition under extended photoperiod regimens in chicken. PeerJ.

[55] Poetzsch C.J., Lewis K., Nicol C.J., Green L.E. (2001). A cross-sectional study of the prevalence of vent pecking in laying hens in alternative systems and its associations with feather pecking, management and disease. Appl. Anim. Behav. Sci..

[56] Ma H., Xin H., Zhao Y., Li B., Shepherd T.A., Alvarez I. (2016). Assessment of lighting needs by W-36 laying hens via preference test. Animal.

[57] Lewis P.D., Morris T.R., Perry G.C. (1998). A model for the effect of constant photoperiods on the rate of sexual maturation in pullets. Br. Poult. Sci..

[58] Leeson S., Caston L.J., Summers J.D. (2003). Performance of layers given two-hour midnight lighting as growing pullets. J. Appl. Poult. Res..

[59] Leeson S., Caston L.J., Summers J.D. (2003). Potential for midnight lighting to influence development of growing leghorn pullets. J. Appl. Poult. Res..

[60] Chew J.A., Widowski T., Herwig E., Shynkaruk T., Schwean-Lardner K. (2021). The Effect of Light Intensity, Strain, and Age on the Behavior, Jumping Frequency and Success, and Welfare of Egg-Strain Pullets Reared in Perchery Systems. Animals.

[61] Renema R.A., Robinson F.E., Feddes J.J.R., Fasenko G.M., Zuidhoft M.J. (2001). Effects of light intensity from photostimulation in four strains of commercial egg layers: 2. Egg production parameters. Poult. Sci..

[62] Kadir E., Sarıca M., Moise N., Dur M., Aslan R. (2021). Effect of light intensity and stocking density on the performance, egg quality, and feather condition of laying hens reared in a battery cage system over the first laying period. Trop. Anim. Health Prod..

[63] England A., Ruhnke I. (2020). The influence of light of different wavelengths on laying hen production and egg quality. World’s Poult. Sci. J..

[64] Liu K., Xin H., Settar P. (2018). Effects of light-emitting diode light v. fluorescent light on growing performance, activity levels and well-being of non-beak-trimmed W-36 pullets. Animal.

[65] Wei Y., Zheng W., Li B., Tong Q., Shi H. (2020). Effects of a two-phase mixed color lighting program using light-emitting diode lights on layer chickens during brooding and rearing periods. Poult. Sci..

[66] Hassan M.R., Sultana S., Ho Sung C., Ryu K.S. (2013). Effect of Monochromatic and Combined Light Colour on Performance, Blood Parameters, Ovarian Morphology and Reproductive Hormones in Laying Hens. Ital. J. Anim. Sci..

[67] Foster R.G., Follett B.K. (1985). The involvement of a rhodopsin-like photopigment in the photoperiodic response of the Japanese quail. J. Comp. Physiol. A.

[68] Sultana S., Hassan M.R., Choe H.S., Ryu K.S. (2013). The effect of monochromatic and mixed LED light colour on the behaviour and fear responses of broiler chicken. Avian Biol. Res..

[69] Li G., Li B., Zhao Y., Shi Z., Liu Y., Zheng W. (2019). Layer pullet preferences for light colors of light-emitting diodes. Animal.

[70] Li G., Ji B., Li B., Shi Z., Zhao Y., Dou Y.J. (2020). Assessment of layer pullet drinking behaviors under selectable light colors using convolutional neural network. Comput. Electron. Agric..

[71] Shi H., Li B., Tong Q., Zheng W., Zeng D., Feng G. (2019). Effects of LED Light Color and Intensity on Feather Pecking and Fear Responses of Layer Breeders in Natural Mating Colony Cages. Animals.

[72] Lind O., Kelber A. (2009). Avian colour vision: Effects of variation in receptor sensitivity and noise data on model predictions as compared to behavioural results. Vis. Res..

[73] Nicol C.J., Bestman M., Gilani A.M., De Haas E.N., De Jong I.C., Lambton S. (2013). The prevention and control of feather pecking: Application to commercial systems. World’s Poult. Sci. J..

[74] Osorio D., Vorobyev M., Jones C.D. (1999). Colour vision of domestic chicks. J. Exp. Biol..

[75] Wichman A., De Groot R., Håstad O., Wall H., Rubene D. (2021). Influence of Different Light Spectrums on Behaviour and Welfare in Laying Hens. Animals.

[76] Bestman M., Koene P., Wagenaar J.P. (2009). Influence of farm factors on the occurrence of feather pecking in organic reared hens and their predictability for feather pecking in the laying period. Appl. Anim. Behav. Sci..

[77] Drake K.A., Donnelly C.A., Dawkins M.S. (2010). Influence of rearing and lay risk factors on propensity for feather damage in laying hens. Br. Poult. Sci..

[78] Spindler B., Weseloh T., Eßer C., Freytag S.K., Klambeck L., Kemper N. (2020). The Effects of UV-A Light Provided in Addition to Standard Lighting on Plumage Condition in Laying Hens. Animals.

[79] Shimmura T., Suzuki T., Hirahara S., Eguchi Y., Uetake K., Tanaka T. (2008). Pecking behaviour of laying hens in single-tiered aviaries with and without outdoor area. Br. Poult. Sci..

[80] Maddocks S.A., Cuthill I.C., Goldsmith A.R., Sherwin C.M. (2001). Behavioural and physiological effects of absence of ultraviolet wavelengths for domestic chicks. Anim. Behav..

[81] Prescott N.B., Wathes C.M., Jarvis J.R. (2003). Light, vision and the welfare of poultry. Anim. Welf..

[82] Barrett J., Rayner A.C., Gill R., Willings T.H., Bright A. (2014). Smothering in UK free-range flocks. Part 1: Incidence, location, timing and management. Vet. Rec..

[83] Richards G.J., Brown S.N., Booth F., Toscano M.J., Wilkins L.J. (2012). Panic in free-range laying hens. Vet. Rec..

[84] Campo J.L., María García G., Sara García D.V. (2014). Comparison of the tonic immobility duration, heterophil to lymphocyte ratio, and fluctuating asymmetry of chicks reared with or without a broody hen, and of broody and non-broody hens. Appl. Anim. Behav. Sci..

[85] Roden C., Wechsler B. (1998). A comparison of the behaviour of domestic chicks reared with or without a hen in enriched pens. Appl. Anim. Behav. Sci..

[86] Riber A.B., Nielsen B.L., Ritz C., Forkman B. (2007). Diurnal activity cycles and synchrony in layer hen chicks (*Gallus gallus domesticus*). Appl. Anim. Behav. Sci..

[87] Riber A.B., Wichman A., Braastad B.O., Forkman B. (2007). Effects of broody hens on perch use, ground pecking, feather pecking and cannibalism in domestic fowl (*Gallus gallus domesticus*). Appl. Anim. Behav. Sci..

[88] Perre Y., Wauters A.M., Richard-Yris M.A. (2002). Influence of mothering on emotional and social reactivity of domestic pullets. Appl. Anim. Behav. Sci..

[89] Riber A.B., Guzman D.A. (2017). Effects of different types of dark brooders on injurious pecking damage and production-related traits at rear and lay in layers. Poult. Sci..

[90] Riber A.B., Guzman D.A. (2016). Effects of Dark Brooders on Behavior and Fearfulness in Layers. Animal.

[91] Rodenburg T.B., Komen H., Ellen E.D., Uitdehaag K.A., van Arendonk J.A.M. (2008). Selection method and early-life history affect behavioural development, feather pecking and cannibalism in laying hens: A review. Appl. Anim. Behav. Sci..

[92] Jensen A.B., Palme R., Forkman B. (2006). Effect of brooders on feather pecking and cannibalism in domestic fowl (*Gallus gallus domesticus*). Appl. Anim. Behav. Sci..

[93] Gilani A.M., Knowles T.G., Nicol C.J. (2012). The effect of dark brooders on feather pecking on commercial farms. Appl. Anim. Behav. Sci..

[94] Chi Q., Chi X., Hu X., Wang S., Zhang H., Li S. (2018). The effects of atmospheric hydrogen sulfide on peripheral blood lymphocytes of chickens: Perspectives on inflammation, oxidative stress and energy metabolism. Environ. Res..

[95] David B., Mejdell C., Michel V., Lund V., Moe R.O. (2015). Air Quality in Alternative Housing Systems may have an Impact on Laying Hen Welfare. Part II—Ammonia. Animals.

[96] Roque K., Shin K.-M., Jo J.-H., Kim H.-A., Heo Y. (2015). Relationship between chicken cellular immunity and endotoxin levels in dust from chicken housing environments. J. Vet. Sci..

[97] Jiang J., Stevenson D.S., Uwizeye A., Tempio G., Sutton M.A. (2021). A climate-dependent global model of ammonia emissions from chicken farming. Biogeosciences.

[98] Shepherd T.A., Xin H., Stinn J.P., Hayes M.D., Zhao Y., Li H. (2017). Ammonia and Carbon Dioxide Emissions of Three Laying-Hen Housing Systems as Affected by Manure Accumulation Time. Trans. ASABE.

[99] Zhao Y., Shepherd T.A., Li H., Xin H. (2015). Environmental assessment of three egg production systems—Part I: Monitoring system and indoor air quality. Poult. Sci..

[100] German G.S., Karlis P. (2020). Developing EU Environmental Standards for the Food, Drink and Milk Industries: Key Environmental Issues and Data Collection.

[101] Matheus D.O., Fernanda C.S., Jair O.S., Arele A.C., Ilda Fátima Ferreira T., Antônio P.S.C. (2021). Ammonia Emission in Poultry Facilities: A Review for Tropical Climate Areas. Atmosphere.

[102] Nimmermark S., Lund V., Gustafsson G., Eduard W. (2009). Ammonia, dust and bacteria in welfare-oriented systems for laying hens. Ann. Agric. Environ. Med..

[103] Donham K.J., Cumro D., Reynolds S.J., Merchant J.A. (2000). Dose-response relationships between occupational aerosol exposures and cross-shift declines of lung function in poultry workers: Recommendations for exposure limits. J. Occup. Environ. Med..

[104] Mahardhika B.P., Mutia R., Ridla M. (2021). Effort to reduce ammonia gas in the broiler chicken excreta with the addition of probiotic as substitute for antibiotic growth promoter. IOP Conf. Ser. Earth Environ. Sci..

[105] Such N., Csitári G., Stankovics P., Wágner L., Koltay I.A., Farkas V. (2021). Effects of Probiotics and Wheat Bran Supplementation of Broiler Diets on the Ammonia Emission from Excreta. Animals.

[106] Sharma N.K., Choct M., Wu S., Swick R.A. (2017). Nutritional effects on odour emissions in broiler production. World’s Poult. Sci. J..

[107] Zheshi K., Jie C., Xiangjie Z., Xianli L. (2014). Study Progress on Animal Feces Treatment by Microorganism. Meteorol. Environ. Res..

[108] Vela-Aparicio D., Forero D.F., Hernández M.A., Brandão Pedro F.B., Cabeza I.O. (2021). Simultaneous biofiltration of H_2_S and NH_3_ using compost mixtures from lignocellulosic waste and chicken manure as packing material. Environ. Sci. Pollut. Res. Int..

[109] Redding M.R. (2013). Bentonite can decrease ammonia volatilisation losses from poultry litter: Laboratory studies. Anim. Prod. Sci..

[110] Brantsæter M., Nordgreen J., Rodenburg T.B., Tahamtani F.M., Popova A., Janczak A.M. (2016). Exposure to Increased Environmental Complexity during Rearing Reduces Fearfulness and Increases Use of Three-Dimensional Space in Laying Hens (*Gallus gallus domesticus*). Front. Vet. Sci..

[111] Zidar J., Campderrich I., Jansson E., Wichman A., Winberg S., Keeling L. (2018). Environmental complexity buffers against stress-induced negative judgement bias in female chickens. Sci. Rep..

[112] Yan C., Hartcher K., Liu W., Xiao J., Xiang H., Wang J., Zhao X. (2020). Adaptive response to a future life challenge: Consequences of early-life environmental complexity in dual-purpose chicks. J. Anim. Sci..

[113] Vestergaard K.S., Kruijt J.P., Hogan J.A. (1993). Feather pecking and chronic fear in groups of red junglefowl: Their relations to dustbathing, rearing environment and social status. Anim. Behav..

[114] Murillo A.C., Abdoli A., Blatchford R.A., Keogh E.J., Gerry A.C. (2020). Parasitic mites alter chicken behaviour and negatively impact animal welfare. Sci. Rep..

[115] Mens A.J.W., van Krimpen M.M., Kwakkel R.P. (2020). Nutritional approaches to reduce or prevent feather pecking in laying hens: Any potential to intervene during rearing?. World’s Poult. Sci. J..

[116] Ross M., Rausch Q., Vandenberg B., Mason G. (2020). Hens with benefits: Can environmental enrichment make chickens more resilient to stress?. Physiol. Behav..

[117] Martin C.D., Mullens B. (2012). Housing and dustbathing effects on northern fowl mites (*Ornithonyssus sylviarum*) and chicken body lice (*Menacanthus stramineus*) on hens. Med. Vet. Entomol..

[118] Huber-eicher B., Wechsler B. (1998). The effect of quality and availability of foraging materials on feather pecking in laying hen chicks. Anim. Behav..

[119] Tahamtani F.M., Brantsaeter M., Nordgreen J., Sandberg E., Hansen T.B., Nodtvedt A. (2016). Effects of litter provision during early rearing and environmental enrichment during the production phase on feather pecking and feather damage in laying hens. Poult. Sci..

[120] Brantsæter M., Tahamtani F.M., Nordgreen J., Sandberg E., Hansen T.B., Rodenburg T.B. (2017). Access to litter during rearing and environmental enrichment during production reduce fearfulness in adult laying hens. Appl. Anim. Behav. Sci..

[121] Schreiter R., Damme K., von Borell E., Vogt I., Klunker M., Freick M. (2019). Effects of litter and additional enrichment elements on the occurrence of feather pecking in pullets and laying hens—A focused review. Vet. Med. Sci..

